# Desensitization and immune tolerance induction in children with severe factor IX deficiency; inhibitors and adverse reactions to replacement therapy: a case-report and literature review

**DOI:** 10.1186/s13052-015-0116-8

**Published:** 2015-02-19

**Authors:** Andrea Bon, Massimo Morfini, Alessandro Dini, Francesca Mori, Simona Barni, Sottilotta Gianluca, Maurizio de Martino, Elio Novembre

**Affiliations:** Department of Pediatrics, University of Udine, Udine, Italy; Haemophilia Agency, Careggi University Hospital, Florence, Italy; Department of Pediatrics, University of Florence, A. Meyer Children’s University Hospital, Florence, Italy; Allergy Unit, Department of Pediatrics, University of Florence, A. Meyer Children’s University Hospital, Florence, Italy; Haemophilia Centre, “Bianchi-Melacrino-Morelli” Hospital, Reggio Calabria, Italy; Department of Health Sciences, University of Florence, A. Meyer Children’s University Hospital, Florence, Italy

**Keywords:** Children, Desensitization, Inhibitor antibodies, Factor IX deficiency, Immune tolerance induction

## Abstract

Hemophilia B is a rare X-linked recessive disorder with plasma factor IX (FIX) deficiency. 1-3% of patients treated with exogenous FIX-containing products develop inhibitors (i.e. polyclonal high affinity immunoglobulins) that neutralize the procoagulant activity of a specific coagulation factor. Although the incidence of inhibitors in hemophilia B patients is low, most are “high titer” and frequently associated with the development of severe allergic or anaphylactic reactions. Immune tolerance induction as a strategy for inhibitor eradication was first described in 1984. Unfortunately, the overall reported success of immune tolerance induction in FIX deficiency with inhibitors is approximately 25-40%.

We report the case of a 2-year-old boy with hemophilia B severe FIX deficiency (<1%), inhibitor antibodies to FIX development, and a history of adverse reactions to FIX infusions, who underwent a successful desensitization and immune tolerance induction with a daily FIX infusion. With this regimen the inhibitor titer decreased with effective bleeding prevention.

## Background

Among people with hemophilia, approximately 80% have hemophilia A, whereas only 20% have hemophilia B. Hemophilia B is an inherited, X-linked, recessive disorder which results in a deficiency of functional factor IX plasma coagulation. It occurs in approximately one to 30,000 male births, in all populations. Mutations causing this disorder have been found all over the FIX gene located in Xq27.1 [1].

Based on the coagulation factor in the patient’s plasma, hemophilia may be classified as mild (>5%), moderate (1-5%) or severe (<1%). About 30 - 45% of patients with hemophilia B have a severe disease [2], requiring prophylactic or on-demand replacement therapy to prevent major and minor bleeding. The use of highly purified, virally attenuated, plasma-derived coagulation factor products, followed by recombinant factor IX concentrates, lowered the risk of severe bleeding and the transmission of infectious agents, so that the development of inhibitory antibodies is nowadays the most serious complication found in hemophilia B patients [2].

### Inhibitors

An inhibitory antibody is a polyclonal high affinity immunoglobulin that neutralizes the procoagulant activity of a specific coagulation factor. Inhibitor levels are measured using Bethesda Units (BU), and classified as “high titer” (≥5 BU) or “low titer” (<5BU) [2].

Genetics influences the risk associated to the development of inhibitory antibodies. Missense mutations in the FIX gene have almost no risk of inhibitor development [3], whereas large deletions and frame-shift mutations leading to the loss of coding information are much more likely to be associated to it. Large deletions account for only 1–3% of all hemophilia B patients, but are found in 50% of inhibitor patients [1].

It has been postulated that the complete absence of endogenous factor IX protein leads to the induction of inhibitors after exposure to an exogenous factor IX antigen. Associated deletion of neighboring genes can contribute to this phenomenon [4]. Additionally, individuals with complete gene deletions were found to be at greater risk of anaphylaxis. Thus, genetic analysis at birth could be important for identifying those at risk for inhibitors and possible anaphylaxis development.

For determining an inhibitor production risk, immune response genes, environmental factors, and other immune system challenges may play a role [5,6].

The development of inhibitory antibodies is seen in about 30% of patients with severe hemophilia A but only 1-3% of those with hemophilia B [7]. The reason why is unknown, but a structural analogy to other vitamin K-dependent factors may confer some tolerance to FIX. Moreover, approximately 60% of severe hemophilia B results from missense mutations [8], providing an increased proportion of antigenic determinants of FIX and letting the “exogenous” FIX be recognized as itself. The majority of people with hemophilia B who develop inhibitors have a severe disease.

Although the incidence of inhibitors in hemophilia B patients is low, most are “high titer” and frequently associated with the development of severe allergic or anaphylactic reactions, whereas anaphylactic reactions in hemophilia A patients with FVIII inhibitors almost never occur. One hypothesis explaining this difference could be that the smaller FIX molecular weight makes its distribution possible in both intra and extravascular space compared to FVIII, which stays confined to the intravascular space [7]. The extravascular distribution may facilitate mast cell activation and IgE mediated hypersensitivity [2]. Another possible reason is the exposure to higher amounts of exogenous FIX because of the higher than normal concentration in plasma, 5 μg mL^−1^ vs 0,1 μg mL^−1^ of FVIII [2].

Patients with severe hemophilia B are at particular risk for the sudden development of anaphylactic shock or other severe allergic reaction and inhibitor development: while these two events are often closely related temporally, one may precede the other. The development of a FIX inhibitor exposes the patient at greater risk of anaphylaxis with one of his subsequent doses [1].

For the risk of potentially life-threatening reactions it has been suggested that all infants and small children with severe hemophilia B be closely monitored over their first 20 or more infusions with any FIX-containing product in a facility equipped to treat anaphylactic shock [9-11].

Most individuals who develop an inhibitor to FIX do so relatively early in life (within the first 4–5 years), after a median of 9–11 exposure days (EDs) to any FIX-containing product [1].

Data from the international registry organized by Warrier et al. [2] on behalf of the FVIII/FIX Subcommittee of the International Society on Thrombosis and Hemostasis (ISTH)’s Scientific and Standardization Committee (SSC), didn’t find differences in anaphylactic and severe allergic reactions following exposure to intermediate-purity or high-purity (either recombinant or plasma-derived) FIX products.

### Biological mechanisms

IgE-mediated [12] and non-IgE-mediated mechanisms may be involved. IgG1 and IgG4 subclasses were found at the time of anaphylactic reaction in some patients with inhibitors and a history of anaphylaxis [13]. The development of specific IgG inhibitors may subsequently lead to a complete activation of anaphylatoxins and the release of mediators from mast cells [14]. Murine models show that anaphylaxis may occur in an IgE-independent manner, involving specific IgG, FcgRIII, macrophages, basophils, and the platelet-activating factor (PAF) as the major mediator [15].

### Treating patients with inhibitors

Treatment of individuals with FIX deficiency complicated by inhibitors can be divided into two categories: strategies for treatment and/or prevention of acute bleeding and strategies for inhibitor eradication.

Treatment of hemorrhagic episodes in patients with FIX deficiency complicated by inhibitors depends upon the type of bleeding episode, the inhibitor classification (high- vs low-responding, more or less than 5 BU respectively after repeated exposure), and the history and severity of previous infusion reactions.

Administrating FIX to overcome inhibitor titer and achieve hemostatic levels is an option in patients with low-responding inhibitors without previous infusion reactions. Unfortunately, such patients represent a minority.

In patients with high-responding inhibitors and/or previous infusion reactions, the mainstays of treatment (or prevention) of bleeding episodes are activated recombinant factor VII concentrate (rFVIIa) and plasma-derived activated prothrombin complex concentrates (aPCCS). rFVIIa is a recombinant product, and has generally proven to be safe and effective, even if it is expensive and has a short half-life [16,17].

Plasmapheresis or immunoadsorption with staphylococcal protein A may be considered in patients with high inhibitor titers experiencing life-threatening bleeding and are unresponsive to other therapies where using an FIX concentrate would be life- or limb-saving.

### Immune Tolerance Induction (ITI)

ITI as a strategy for inhibitor eradication was first described by Brackmann in 1984 in hemophilia A [18]. There is little data for developing a useful and evidence-based approach to the prevention and eradication of FIX inhibitors [19] due to the small numbers in hemophilia B compared to hemophilia A. Unfortunately, the overall reported success of ITI in FIX deficiency with inhibitors is approximately 25-40% [2].

Often patients with high titer inhibitors have a history of severe allergic reactions and anaphylaxis to FIX infusion (allergic phenotype) and need desensitization with gradually increased doses of FIX prior to ITI. Dioun et al. [12] have described a successful desensitization protocol involving skin testing to FIX-containing products; this was followed by an infusion lasting several hours, and was the base of our desensitization protocol. Some successful desensitization protocols with or without immune modulation have been reported, such as the use of plasmapheresis, or an addition of steroids or rituximab before, during or after the escalating dose [12,20-22].

We report the case of a 2-year-old boy with severe FIX-deficiency (<1%), inhibitor development, and a history of adverse reactions to FIX infusions, who underwent a successful desensitization and ITI.

## Case presentation

We report the case of a 2-year-old boy who was diagnosed with severe FIX deficiency at 9 months of age after testing due to frequent bruising of the skin. The genetic analysis reported a nonsense mutation of the FIX gene. Recombinant FIX (BeneFIX®, rFIX) was started on demand, and the young patient was treated several times without complications. At 1 year of age the patient developed pallor, sweating, agitation and then fainting during rFIX infusion. The patient recovered within minutes after suspending therapy. There was no rash or respiratory distress. The next day the infusion was repeated and the patient exhibited a transient perioral cyanosis and agitation, but therapy was completed without other complications. Blood tests revealed an initial inhibitor titer of 2.7 Bethesda Units (BU), with a peak of 25 BU one month later.

For this reason and for fear of severe adverse reactions, BeneFIX® was stopped and bleeding episodes were treated with a recombinant factor VII concentrate (rFVIIa, NovoSeven®). Response was suboptimal even with high doses (230 μg/kg) with very frequent bleeding.

For the declining quality of life, with the purpose of re-introducing factor IX, the patient was admitted to our center at 23 months of age. Inhibitor titer was 0,1 BU.

The skin prick test with FIX concentrate was negative, with normal saline and histamine used as negative and positive controls, respectively. Intradermal tests with factor IX concentrate in 1:100 and 1:10 dilution were also negative, with a negative control of normal saline. Thus, we performed a challenge test by infusing 30 U/kg of FIX diluted in 50 mL of normal saline for 20 minutes, then 120 U/Kg diluted in 10 mL for 20 minutes without any reaction.

Therefore the dosage of 150 U/Kg was initially maintained daily, then every 2 or 3 days. During his fifth dose he developed a new reaction with agitation, cough, facial hyperemia and then cyanosis, soon after starting the infusion. He was treated with hydrocortisone with a complete resolution of the symptoms. The inhibitor titer was 37 BU and peaked at 60 BU three days later.

The sudden and unexpected reaction with respiratory involvement and the concomitant rise in the inhibitor titer discouraged us from continuing this approach, so we stopped the FIX.

At that point we started a desensitization protocol with increasing FIX doses to allow us to start an ITI regimen (Table 1). After the first dose (0,01 U/kg) the patient manifested agitation, mild facial hyperemia without respiratory involvement. Nevertheless, the desensitization was continued and the subsequent doses were well tolerated without any adverse effects.

**Table 1:** **Factor IX desensitization protocol (modified from Dioun et al.** [12]**)**

	**Dose (U/kg)**	**Cumulative dose (U/kg)**	**Infusion time**	**Interval from previous dose (min)**
Day 1	0.01	0.01	5 min	0
	0.02	0.03	5 min	10
	0.04	0.07	5 min	10
	0.08	0.15	5 min	10
	0.10	0.25	5 min	10
	0.02	0.45	5 min	20
	0.04	0.85	5 min	20
	0.08	1.65	5 min	20
	1.5	3.15	5 min	20
	3	6.15	30 min	-
	6	12.15	30 min	-
	9	21.15	60 min	-
	10	31.15	60 min	-
Day 2	40	40	10 h	-
Day 3	40	40	8 h	-
Day 4	40	40	6 h	-
Day 5	40	40	4 h	-
Day 6	40	40	2 h	-
Day 7	40	40	1 h	-
Day 8	40	40	30 min	-
Day 9	40	40	20 min	-

**Figure 1:**
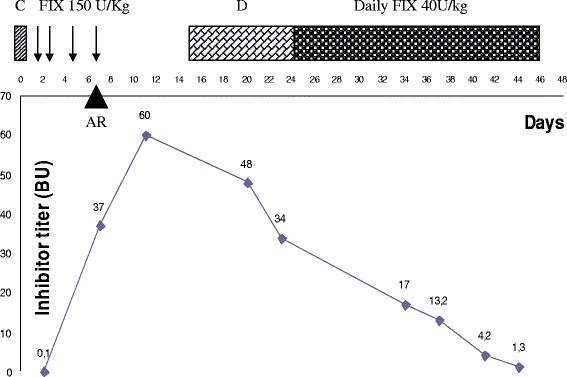
**The inhibitor titer after the challenge, during FIX infusion, desensitization and ITI regimen.** C: Challenge with rFIX; D: desensitization; AR: adverse reaction, BU: Bethesda Unit.

Now the patient is receiving 40 U/kg/day for 20 minutes without reactions, and the inhibitor level is slowly decreasing (Figure 1).

## Conclusion

In this case report our patient with severe haemophilia B developed inhibitors and adverse reactions to the FIX infusion. Biological mechanisms of immune tolerance in hemophilia patients are largely unknown, and derive from studies on hemophilia A. The development of anti-idiotypic antibodies, T-cell anergy and inhibition of memory B cell differentiation possibly play a role [23].

The way the drug is administered is perhaps important in developing inhibitors and tolerance induction. Studies on biological drugs show that patients receiving episodic treatment develop anti-drug-antibodies more frequently than those receiving scheduled therapy [24]. Consistent to this observation, the patient developed an inhibitor and allergic reactions when treated episodically, but with daily scheduled infusion inhibitor titer is decreasing and the therapy is well tolerated.

Desensitization opens the way to an ITI, using daily or larger daily doses of FIX every other days.

In particular, a desensitization protocol allowed us to start ITI with a daily FIX infusion. In our ITI regimen, 40 U/kg (500 U) given once daily were sufficient to decrease the inhibitor titer and to control bleeding. The dose is considerably lower than that used in other published studies [21,22], although it was equally effective.

Even when effective, ITI can present severe complications such as nephrotic syndrome, typically 8–9 months into therapy. It is more frequent in patients with previous infusion reactions to FIX (allergic phenotype). Frequently it is nonresponsive to steroids and requires the suspension of ITI [2]. Renal biopsies demonstrated membranous glomerulonephritis in two patients [25-27]. In this case follow up is not long enough and immune tolerance not achieved yet so it is not possible to know whether the patient would develop nephrotic syndrome.

## Consent

Written informed consent was obtained from the patient for publication of this case report and any accompanying images. A copy of the written consent is available for review by the Editor-in-chief of this journal.

## Abbreviations

FIX: Factor IX
rFIX: Recombinant FIX
BeneFIX®: Recombinant FIX
BU: Bethesda Units
rFVIIa: Recombinant factor VII
NovoSeven®: Recombinant factor VII
ITI: Immune tolerance induction
EDs: Exposure days
ISTH: International Society on Thrombosis and Hemostasis
SSC: Scientific and Standardization Committee
PAF: Platelet-activating factor
aPCCS: Plasma-derived activated prothrombin complex concentrates
